# Chi8: a GPU program for detecting significant interacting SNPs with the Chi-square 8-df test

**DOI:** 10.1186/s13104-015-1392-5

**Published:** 2015-09-14

**Authors:** Abdulrhman Al-jouie, Mohammadreza Esfandiari, Srividya Ramakrishnan, Usman Roshan

**Affiliations:** King Abdullah Medical Research Center, King Saud Bin Abdulaziz University for Health Sciences, P.O. Box 22490, Riyadh, 11426 Saudi Arabia; Department of Computer Science, New Jersey Institute of Technology, GITC 4400, University Heights, Newark, NJ 07102, USA; Cold Spring Harbor Laboratory, 1 Bungtown Road, Cold Spring Harbor, NY 11724 USA

**Keywords:** Graphics Processing Unit, Interacting SNPs, Epistasis, Chi-square 8 degrees of freedom, Genome wide association studies

## Abstract

**Background:**

Determining interacting SNPs in genome-wide association studies is computationally expensive yet of considerable interest in genomics.

**Findings:**

We present a program Chi8 that calculates the Chi-square 8 degree of freedom test between all pairs of SNPs in a brute force manner on a Graphics Processing Unit. We analyze each of the seven WTCCC genome-wide association studies that have about 5000 total case and controls and 400,000 SNPs in an average of 9.6 h on a single GPU. We also study the power, false positives, and area under curve of our program on simulated data and provide a comparison to the GBOOST program. Our program source code is freely available from http://www.cs.njit.edu/usman/Chi8.

**Electronic supplementary material:**

The online version of this article (doi:10.1186/s13104-015-1392-5) contains supplementary material, which is available to authorized users.

## Findings

### Background

Detecting interacting SNPs in a genome-wide association study (GWAS) is a problem of considerable importance in genomics [1, 2]. Many solutions have been proposed that run on a CPU and either examine all pairs of SNPs [3–5] or a smaller set after pruning [6, 7]. The brute force solution of examining each pair in a serial fashion takes months to finish. To speed this up several parallel solutions have been proposed on Graphics Processor Units (GPUs). A GPU can run several hundred threads at the same time and allows for massive parallelism in computer programs (see http://www.gpucomputing.net).

Recent GPU programs in this area include SHEsisEpi [8], EPIBLASTER [9] and GBOOST [10]. Each of these offers different statistics. SHEsisEpi calculates ratios of odds ratios between cases and controls and EPIBLASTER calculates the difference in Pearson correlation between cases and controls. GBOOST has a fast screening phase followed by testing for significant pairs using a log likelihood ratio test. Our goal here is not to compete against these statistics since each has its own strengths and weaknesses.

Instead we present a fast GPU program for calculating the Chi-square 8-df test between all pairs of SNPs. This test has been studied previously [11, 12] in CPU implementations. In the first study [11] it serves as a baseline for comparison and takes hours to finish on much smaller GWAS than the ones we consider in this study. In the latter [12] it is applied only on a subset of pairs of SNPs instead of all pairs. Our approach is also similar to SNPRuler [13] and so from a theoretical perspective we do not offer a new statistical test. However, the runtimes in these studies are still high for brute force search across all pairs of SNPs in large-scale genome-wide association studies [2, 11]. Our implementation of this test runs on a GPU and takes advantage of its massive parallelism.

Our program finishes in an average of 9.6 h on the Wellcome Trust Case Control Consortium (WTCCC) GWAS, that have an average of 4800 case and controls and 400,000 SNPs. On simulated data our program has comparable power to GBOOST [10] (GPU counterpart of BOOST [14]) but much fewer false positives. Both have a comparable area under curve on 1600 subjects but on 800 subjects Chi8 performs better. On real data our program reports interacting SNPs some of which are also found by GBOOST and also supported by the literature. Below we describe our program followed by experimental results.

### Methods

#### Chi8 algorithm

**Fig. 1:**
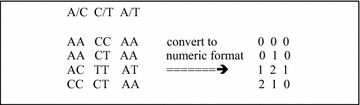
GWAS with four subjects and three SNPs shown on the* left*. On the* right* is the numeric format of the GWAS. For each SNP shown in the* first row* on the* left* we count the number of occurrences of the nucleotide of the larger letter

Our program, that we call Chi8, computes the Chi-square 8-df test between all pairs of SNPs in a parallel. The input to Chi8 is numeric format genome wide association study (GWAS) that we briefly describe here. A GWAS is a matrix of SNPs where each SNP is given by a string of two letters each taking on the values A, C, G, and T. We convert each SNP into ‘0’, ‘1’, and ‘2’ to represent the number of copies of the allele with the larger alphabet value [15, 16]. In the numeric format the GWAS is given by an *n* by *m* matrix of characters taking on the values ‘0’, ‘1’, and ‘2’ where *n* is the number of subjects and *m* is the number of SNPs. We assume that all case subjects appear before controls in the GWAS. In Fig. 1 we show a simple GWAS of four subjects and three SNPs and its numeric format.

**Fig. 2:**
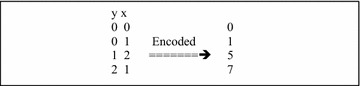
GWAS with four subjects and two SNPs shown on the* left*. On the* right* is the encoded GWAS for the Chi-square 8-df test using the formula $x+3y$

Before describing the Chi8 algorithm we explain how to compute 8-df Chi-square test on two SNPs (as done previously [11, 12]). Let *x* and *y* be the two SNPs that can take on values 0, 1, and 2 each. To apply the 8-df test we first encode them to take on values 0 through 8 through the simple formula $x + 3*y$. In Fig. 2 we show the encoding of a simple GWAS of two SNPs *y* and *x*.

After the encoding we create a $2\times 9$ contingency table to compute the Chi-square p-value. To account for zero entries in the table we use pseudocounts by initializing all entries to one instead of zero.

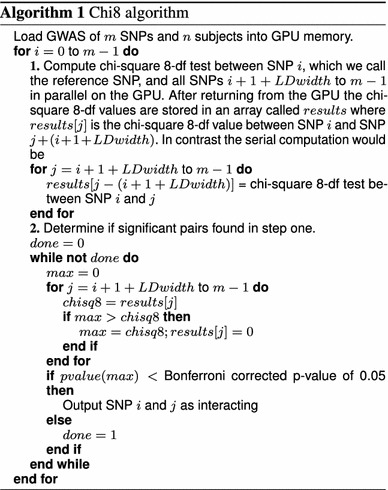



In Algorithm 1 we provide the algorithmic description of Chi8. In the first part we compute in parallel Chi-square 8-df values between a fixed SNP *i* and remaining ones of larger index upto the last one. In the second part we check for p-values that are below the Bonferroni threshold. Instead of sorting the results from part one, which can take considerable time, we simply find the min p-value, set it to 0 in the results, and repeat until the min p-value is above the Bonferroni corrected threshold.

**Fig. 3:**
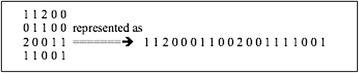
GWAS with four subjects and five SNPs shown on the* left*. On the* right* is the one dimensional representation where each* row* follows the previous one

To obtain high speeds in GPUs it is essential that memory access be coalescent. This means consecutive threads access consecutive memory locations. To achieve this we store the GWAS in a large one-dimensional character array in row first format (see Fig. 3).

We copy the one dimensional GWAS onto the GPU memory just once in the beginning of the program. We then fix a reference SNP (see Algorithm 1) that is accessed by the thread with identifier 0. All SNPs following the reference are accessed by threads 1 through $m-i-1-LDwidth$ where *m* is the total number of SNPs, *i* the reference, and LDwidth is set to 100 by default. The *LDwidth* constraint eliminates pairwise SNPs that are in strong linkage disequilibrium and that usually lie on the same gene.

**Fig. 4:**
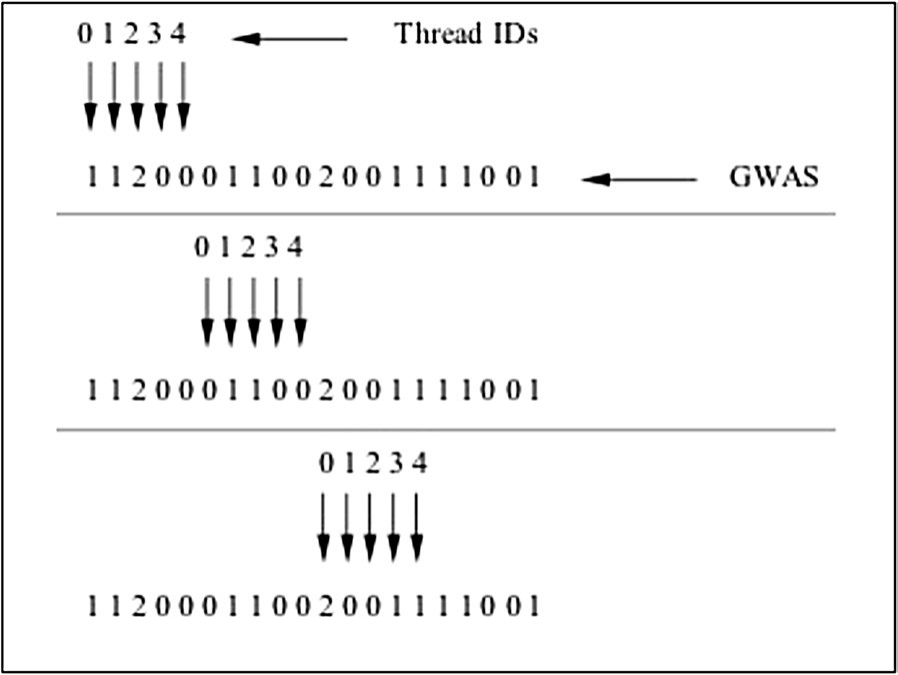
Toy example on a GWAS with four subjects and five SNPs (from Fig. 3) depicting the Chi8 algorithm. Threads 1 through 4 each simultaneously consider the combination of SNPs in columns 1 through 4 with column 0 that is the reference. The thread pointers then incrementally increase by five (the number of columns) to the following row to complete the contingency tables. Note that *LDwidth* is set to 0 in this example

Each thread compares the value of its SNP with the reference, encodes it to an integer between 0 and 8 [11, 12], and updates the $2\times 9$ contingency table. For the next row all thread pointers move ahead by *columns* and the counting continues (see Fig. 4). Thus, for a fixed reference column *i* all pairwise combinations with following SNPs are computed in parallel and outputted if the Chi-square 8-df p-value is below the Bonferroni corrected threshold. The reference column then increases by one and the procedure is repeated until the reference reaches the second last column.

#### Sensitivity of Chi8 to univariate significant SNPs

**Fig. 5:**
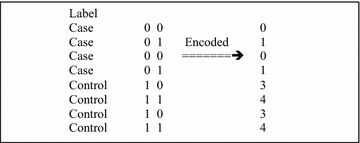
Toy example showing the effect of univariate significant SNPs on the Chi8 encoding. If either one of the SNPs is significant on its own, as shown in this example, the resulting encoding is also likely to be significant. If we quadruple the number of case and controls in this example our program Chi8 returns a p-value of 0.00123 (keeping in mind that it initializes the contingency table with one’s instead of zero’s to avoid divide by zero—a common heuristic).

Due to the nature of our encoding the program Chi8 will tend to output pairs of SNPs if one is significant on its own. For example consider the two SNPs shown in Fig. 5. One is highly significant while the other is not at all. After the encoding we see that the two pairs are likely to be reported as significant since the case group has just 0’s and 1’s whereas the control has 3’s and 4’s. If we quadruple the number of cases and controls our program Chi8 outputs a p-value of 0.00123. Note that we use pseudocounts and initialize all entries of the contingency table to one instead of zero.

#### Datasets

We consider genome-wide association studies for seven diseases from the Wellcome Trust Case Control Consortium (WTCCC). We followed standard protocols for cleaning the data [17]. We removed SNPs that deviated significantly from the Hardy–Weinberg equilibrium and SNPs with greater than 1 % missing entries (Table 1).

We also study publicly available simulated datasets used in the BOOST study [14]. These are four models each with a total of 800 and 1600 cases and controls (equal proportion) and mean allele frequencies of 0.1, 0.2, and 0.4. Under each setting there are 100 simulated datasets each containing 1000 SNPs where the first and last are interacting and also have main effects. In brief Model 1 is a multiplicative model, Model 2 is an epistasis model used to describe handedness and the color of swine, Model 3 is the classical epistasis model, and Model 4 is the popular exclusive OR (XOR) model. More details about the models and their simulation can be found in the BOOST study [14]. We downloaded these datasets from the BOOST website http://bioinformatics.ust.hk/BOOST.html.

### Results

#### Experimental settings

We ran both Chi8 and GBOOST on the real and simulated datasets on Intel Xeon E5-2660v2 machines with 64 GB RAM and NVIDIA K20X GPUs with 6 GB RAM. On simulated data we set the LDwidth of Chi8 to zero and on real data we use an LDwidth of 100. We ran GBOOST with a screen threshold (BOOST interaction threshold) of 37. We obtained this value by starting from the default in the program (of 30) and increasing it until the power was equal to previously published values on model 1 allele frequency of 0.1 [14]. Otherwise with the default parameter GBOOST outputs a considerably high number of false positives (in the range of 0.98–1 for all model settings).

#### Measure of accuracy and error

The power is defined as the fraction of 100 simulated datasets in each setting where the true interacting pair is significant under the given test. We define the false positive rate as the fraction of 100 simulated datasets where at least one interacting SNP is reported that is not the true interaction. We define the mean area under curve as the area under curve averaged across the 100 datasets.

#### Simulated data

In Tables 2 and 3 we show the power and false positive rates of the two programs. To account for Chi8’s sensitivity to univariate significant SNPs we only consider reported pairs in the output where both SNPs are individually insignificant. In the simulated data this corresponds to a Bonferroni corrected p-value of 0.05/1000.

**Table 1:** WTCCC datasets that are used in our study

Data	Cases	Controls	SNPs
Type 1 diabetes (T1D)	1963	2938	422,006
Rheumatoid arthritis (RA)	1860	2938	403,301
Crohn’s disease (CD)	1748	2938	405,306
Type 2 diabetes (T2D)	1924	2938	402,532
Hypertension (HT)	1952	2938	402,895
Bipolar disorder (BD)	1868	2938	396,320
Coronary artery disease (CAD)	1926	2938	404,145

**Table 2:** Power and false positive rates of Chi8 and GBOOST on simulated GWAS of 800 subjects

Allele freq.	0.1	0.2	0.4
Model 1			
Chi8	0 (0)	0.02 (0.01)	0.01 (0.01)
GBOOST	0.02 (0.08)	0.01 (0.07)	0 (0.07)
Model 2			
Chi8	0.16 (0)	0.55 (0.01)	0.49 (0)
GBOOST	0 (0.08)	0.61 (0.07)	0.53 (0.06)
Model 3			
Chi8	0 (0)	0.1 (0.01)	0.18 (0)
GBOOST	0.01 (0.13)	0.06 (0.06)	0.5 (0.09)
Model 4			
Chi8	0.18 (0)	0.44 (0)	0.55 (0)
GBOOST	0.01 (0.08)	0.42 (0.11)	0.78 (0.09)

To account for Chi8’s sensitivity to univariate significant SNPs we only consider reported pairs where both SNPs are also individually insignificant

**Table 3:** Power and false positive rates of Chi8 and GBOOST on simulated GWAS of 1600 subjects

Allele freq.	0.1	0.2	0.4
Model 1			
Chi8	0.22 (0.02)	0.04 (0)	0 (0)
GBOOST	0.45 (0.12)	0.37 (0.08)	0.09 (0.05)
Model 2			
Chi8	0.22 (0.01)	0.94 (0.02)	0.91 (0.01)
GBOOST	0.32 (0.09)	0.98 (0.04)	0.98 (0.1)
Model 3			
Chi8	0.25 (0.02)	0.36 (0.02)	0.77 (0)
GBOOST	0.23 (0.11)	0.49 (0.11)	0.96 (0.06)
Model 4			
Chi8	0.09 (0)	0.73 (0.02)	1 (0.02)
GBOOST	0.29 (0.08)	0.98 (0.13)	0.98 (0.18)

To account for Chi8’s sensitivity to univariate significant SNPs we only consider reported pairs where both SNPs are also individually insignificant

With 800 subjects Chi8 has higher power in half of the settings whereas GBOOST in the other half. With 1600 subjects GBOOST has higher power in 10 out of 12 settings and Chi8 in 2 settings. However, when comparing false positives Chi8 performs better in all 12 model settings with both 800 and 1600 subjects. In models 3 and 4 the power of both methods increases with mean allele frequencies whereas in model 1 (multiplicative) the power decreases for both methods.

These results are subject to the p-value and interaction score thresholds of Chi8 and GBOOST respectively. If we use the default threshold score of 30 in GBOOST we obtain far more false positives. The power and false positive rates reported here are for a threshold of 37 that yields similar power as previously published [14]. For Chi8 we use the standard 0.05 Bonferroni corrected p-value as the threshold.

In Tables 4 and 5 we show the mean area under curve of Chi8 and GBOOST respectively. To compute the area under curve we would need true and false positive rates for different thresholds of the two programs. For Chi8 we considered p-value thresholds of 1, 5E–6, 3E–6, 1E–6, 5E–7, 1E–7, 5E–8, 1E–8, 5E–9, 1E–9, 5E–10, 1E–10, 5E–11, 1E–11, and 1E–29. For GBOOST we used thresholds of 0, 30, 33, 35, 37, 38, 40, 50, 60, 70, 80, and 100. We see that both are competitive. In 800 subjects Chi8 has a better mean area under curve of 0.64 vs. 0.34 for GBOOST. In 1600 GBOOST is slightly better: Chi8 has a mean of 0.64 and GBOOST of 0.67. In the Additional file 1 we provide the mean ROC curve for both Chi8 and GBOOST on all model settings.

**Table 4:** Mean area under curve of Chi8 and GBOOST on simulated GWAS of 800 subjects

Allele freq.	0.1	0.2	0.4
Model 1			
Chi8	0.4	0.41	0.32
GBOOST	0.1	0.1	0.07
Model 2			
Chi8	0.68	0.88	0.85
GBOOST	0.08	0.64	0.68
Model 3			
Chi8	0.45	0.54	0.81
GBOOST	0.09	0.14	0.62
Model 4			
Chi8	0.64	0.84	0.86
GBOOST	0.09	0.54	0.86

To account for Chi8’s sensitivity to univariate significant SNPs we only consider reported pairs where both SNPs are also individually insignificant

**Table 5:** Mean area under curve of Chi8 and GBOOST on simulated GWAS of 1600 subjects

Allele freq.	0.1	0.2	0.4
Model 1			
Chi8	0.56	0.15	0.04
GBOOST	0.43	0.54	0.22
Model 2			
Chi8	0.46	0.98	0.97
GBOOST	0.49	0.98	0.98
Model 3			
Chi8	0.68	0.65	0.93
GBOOST	0.37	0.62	0.97
Model 4			
Chi8	0.35	0.89	1
GBOOST	0.43	0.98	0.98

To account for Chi8’s sensitivity to univariate significant SNPs we only consider reported pairs where both SNPs are also individually insignificant

#### Real data

In Table 6 we show the total time for Chi8 and GBOOST to run on the real datasets. We see that Chi8 finishes in an average of 9.6 h on the WTCCC datasets. Except for type 1 diabetes the runtimes are similar for the other datasets. This disease has the most number of SNPs and contains the most significant pairs and so has the highest runtime. GBOOST in comparison has a faster runtime.

**Table 6:** Total Chi8 and GBOOST time in hours to run on the seven WTCCC GWAS dataset

Data	T1D	RA	CD	CAD	T2D	HT	BD
Time							
Chi8	14.2	10.1	8.4	8.6	8.6	8.7	8.3
GBOOST	1.7	1.6	1.6	1.6	1.6	1.6	1.5
Number of significant pairs							
Chi8	1644	11	4	0	0	0	0
GBOOST	28K	11K	9.9K	10K	10K	10K	9.8K
GBOOST*	92	0	0	0	0	0	0
BOOST (published [14])	91	0	1	0	0	0	0
Number of common pairs between Chi8 and GBOOST							
	236	2	4	NA	NA	NA	NA

We also show number of significant pairs outputted by each program such that both SNPs are insignificant on their own. Otherwise our program Chi8 outputs thousands of interactions where one SNP is significant on its own. For GBOOST we use the same interaction threshold of 37 as we did for the simulated data. We also constrain the GBOOST output (denoted as GBOOST*) to consider just pairs with a threshold above 175 and at least 100 SNPs apart to account for linkage. We selected this threshold so as to output a similar number of pairs for type 1 diabetes as published in the original BOOST paper [14]. The previously published values from the original BOOST study consider pairs that are at least 1MB apart and individually insignificant [14]

To estimate the speedup against a serial version we wrote a simple C program for calculating Chi-square 2-df test p-values on a GWAS. This finished in 400 seconds on the Crohn’s disease dataset that has slightly above 400,000 SNPs and 4686 case and controls. If we estimate the time for doing all $400,000\atopwithdelims ()2$ pairs serially it takes at least two years. Our program Chi8 in comparison finishes under 9 h on this dataset.

We found several significant interacting SNPs in our datasets at least the LDwidth distance (of 100 SNPs by default). We only report number of pairs such that each SNP is individually insignificant. In Table 6 we order the datasets from left to right in decreasing order by the number of Chi-square 2-df significant SNPs found in the dataset. We see that the number of reported pairs are also in decreasing order given Chi8’s sensitivity to univariate significant SNPs (as described earlier). We provide details of all pairs including dbSNP identifiers [18], individual 2-df, and pairwise 8-df p-values in individual text files on the website http://www.cs.njit.edu/usman/Chi8.

*Type 1 diabetes* In type 1 diabetes all of our reported SNP interactions lie on chromosome 6 starting from position 26.35 MB and ending at position 33.16 MB in human genome reference GRCh37.p13. This region contains genes from the HLA complex that is well known to be associated with type 1 diabetes [17]. SNPs in this region are also known to be in high linkage disequilibrium [19] and so we see many reported interactions.

*Arthritis* In arthritis all of our reported interactions except for one also lie on chromosome 6 starting at position 31.3 MB and ending at 32.9 MB. This region is also known to contain SNPs in high linkage disequilibrium [20]. Outside of this region we find an interaction between a SNP on chromosome 1 at position 82.4MB and on chromosome 6 at position 32.8 MB near the HLA-DOB gene. The SNP on chromosome 1 is near the CYR61 gene (at position 85 MB) that is known to be associated with arthritis [21]. This SNP is also not too far from the PTPN22 gene (113.8 MB) that is known to have interactions with genes in the HLA region on chromosome 6 [22] like we have reported.

*Crohn’s disease* Here we find interactions between a SNP in the IL23R gene and four consecutive SNPs on chromosome 11 at position 76.3 MB. While IL23R is well known to be associated with Crohn’s disease [23] this reported interaction is not previously studied.

Both Chi8 and GBOOST report several common SNP interactions in each of the three diseases. In Crohn’s disease GBOOST also reports the four interactions outputted by Chi8. In type 1 diabetes and arthritis both programs report interactions in the HLA region of genes.

### Discussion

Most methods for predicting disease risk rely on SNPs detected by univariate tests. To evaluate the predictive power of our reported significant pairs we performed two tests. First we computed the Pearson correlation coefficient between the encoded pair of SNPs and classification labels (0 to denote case and 1 to denote control). In Crohn’s disease we considered the most significant pair of rs4655684 and rs12789493 and determined its Pearson coefficient to be 0.092. We also considered the single significant pair in arthritis across chromosomes 1 and 6 and found its Pearson coefficient to be 0.034. Both are low to be of prediction utility.

Second, we determined the risk prediction accuracy of the interacting SNPs using the support vector machine which is a popular state of the art classifier [24]. In Crohn’s disease and arthritis we considered the predicted pairs in addition to individually significant SNPs in tenfold cross-validation study [24]. We found it yielded a marginal change in accuracy if individually significant SNPs were used on their own. This held true even if we explicitly considered interactions under the degree 2 polynomial kernel for the support vector machine. Similarly, reported SNP interactions in type 1 diabetes were also used for prediction and did not yield a higher accuracy [19].

We also notice that our method is a more stringent test than the LD-contrast implemented in the SIXPAC [5] program. For example SIXPAC reports the SNPs rs10925490 and rs2041140 as significant in the WTCCC bipolar disorder dataset with a p-value of $4.61 \times 10^{-14}$. On the other hand the Chi-square 8-df p-value of this pair is $4.57 \times 10^{-8}$ which is insignificant under the Bonferroni correction and thus not reported by our program. As above, this pair also has a low Pearson correlation coefficient of 0.002 against the classification labels (0 for case and 1 for control).

### Conclusion

Our program Chi8 offers a fast solution to computing the Chi-square 8-df test between all pairs of SNPs in large genome-wide association studies.

## Availability and requirements

*Project name* Chi8.

*Project home page*http://www.cs.njit.edu/usman/Chi8.

*Operating system* Linux (tested on Red Hat Enterprise Linux 6.2).

*Programming language* C and CUDA (version 4.2 or greater). The latter is the NVIDIA language for their GPUs. For C we used gcc version 4.4.6 (Red Hat 4.4.6-3).

*License* Please contact authors for commercial use. Academic use is free.

## Availability of supporting data

Our real data is available directly by request from the Wellcome Trust Case Control Consortium and the National Institute of Health database of genotypes and phenotypes. The data access agreements prohibit us from posting this data publicly. The simulated data is freely available from the BOOST website http://bioinformatics.ust.hk/BOOST.html and the Chi8 source code from http://www.cs.njit.edu/usman/Chi8.

## Additional file

**Additional file 1.** Mean ROC curves for Chi8 and GBOOST on all model settings.
