# Supplementary material for: Chi8: a GPU program for detecting significant interacting SNPs with the Chi-square 8-df test
Source: BMC Res Notes. 2015 Sep 14;8:436. doi: 10.1186/s13104-015-1392-5 (PMC4568583; doi:10.1186/s13104-015-1392-5)
Supplement: Supplementary file 1 — Additional file 1. Mean ROC curves for Chi8 and GBOOST on all model settings. [file 13104_2015_1392_MOESM1_ESM.pdf]

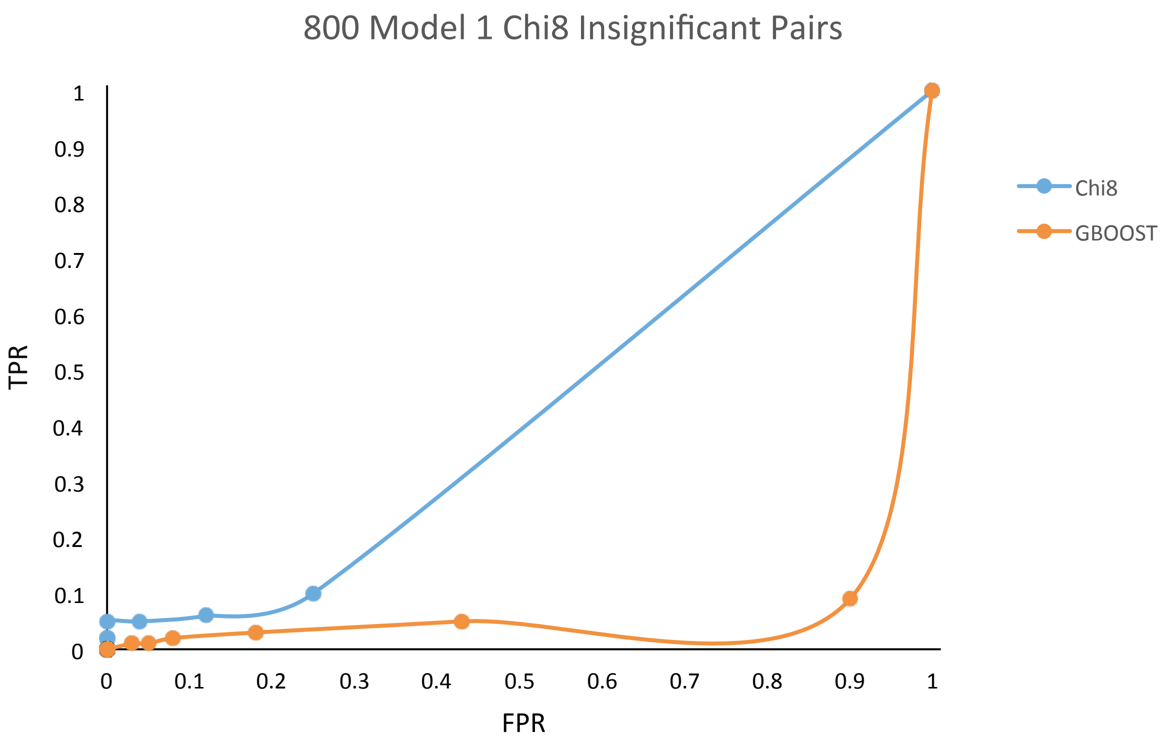
800 Model 1 Chi8 Insignificant Pairs
TPR
FPR
0
0.1
0.2
0.3
0.4
0.5
0.6
0.7
0.8
0.9
1
Chi8
GBOOST

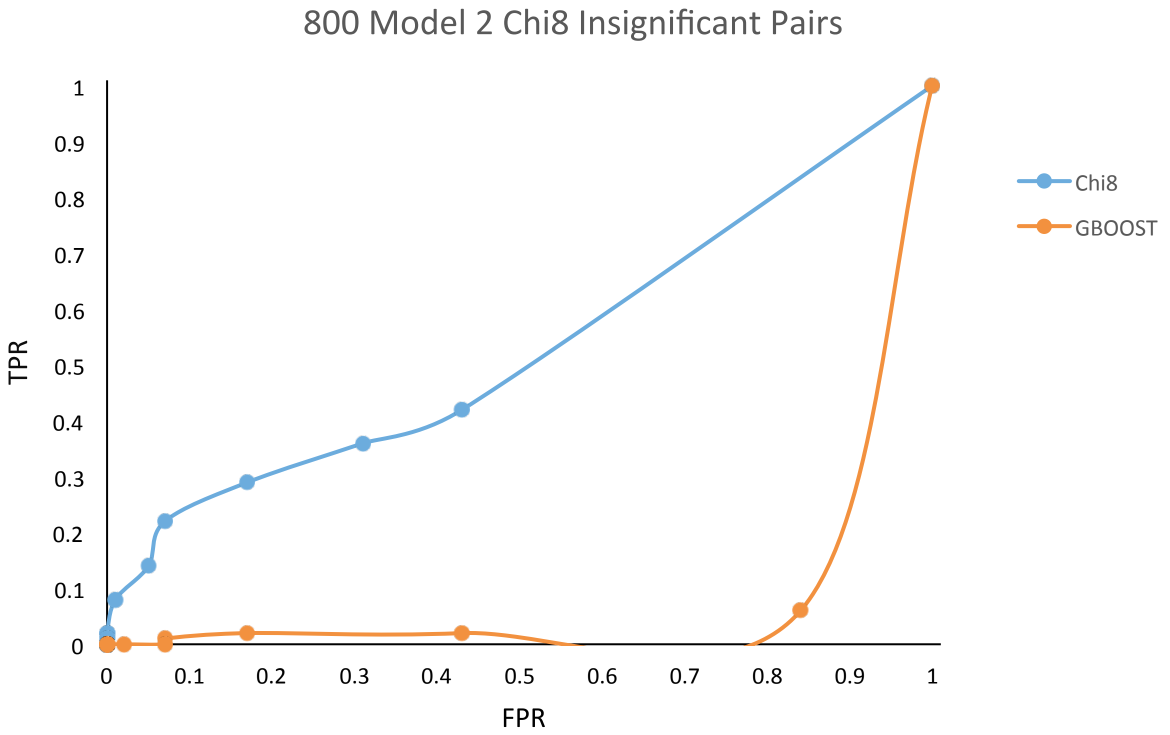

800 Model 2 Chi8 Insignificant Pairs
TPR
FPR
0
0.1
0.2
0.3
0.4
0.5
0.6
0.7
0.8
0.9
1
Chi8
GBOOST

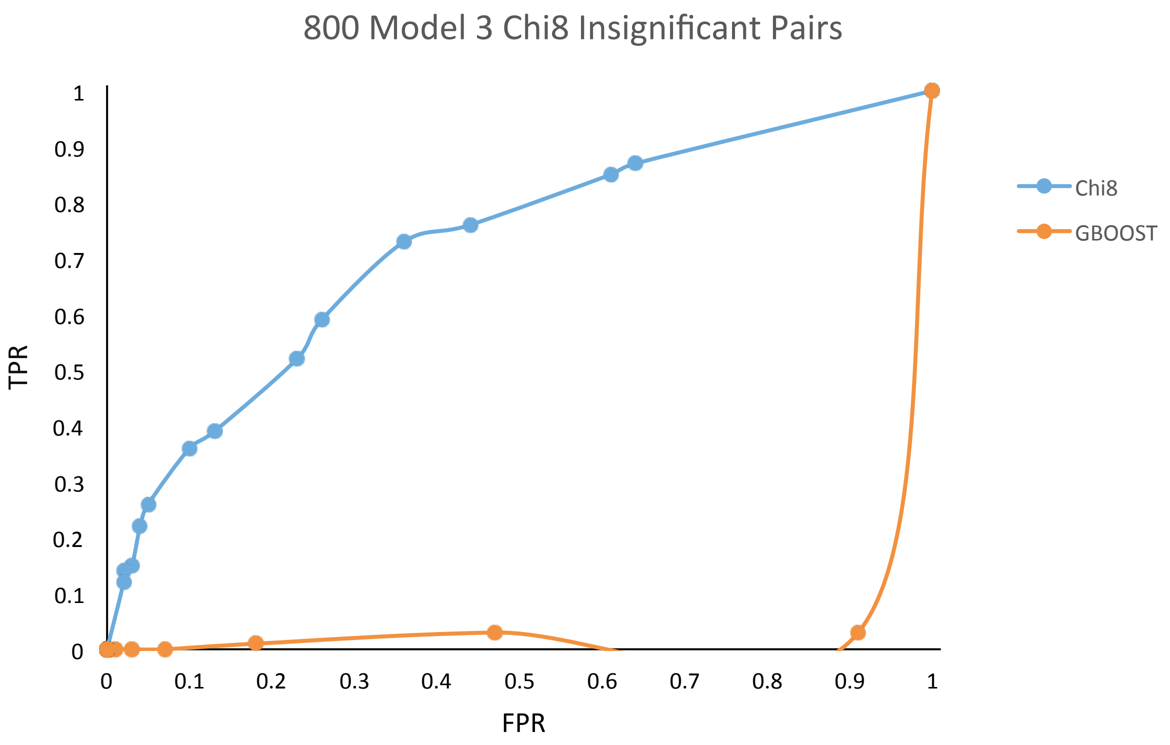

800 Model 3 Chi8 Insignificant Pairs
TPR
FPR
0
0.1
0.2
0.3
0.4
0.5
0.6
0.7
0.8
0.9
1
Chi8
GBOOST

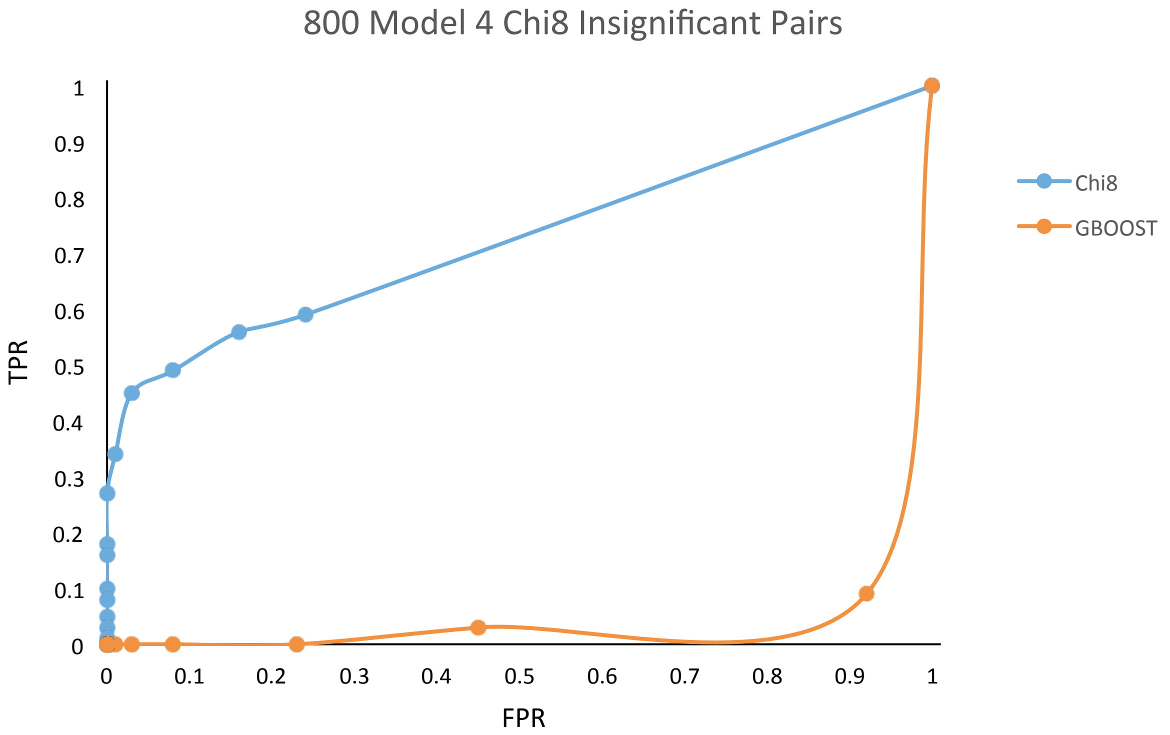
800 Model 4 Chi8 Insignificant Pairs
TPR
1
0.9
0.8
0.7
0.6
0.5
0.4
0.3
0.2
0.1
0
0
0.1
0.2
0.3
0.4
0.5
0.6
0.7
0.8
0.9
1
FPR
Chi8
GBOOST

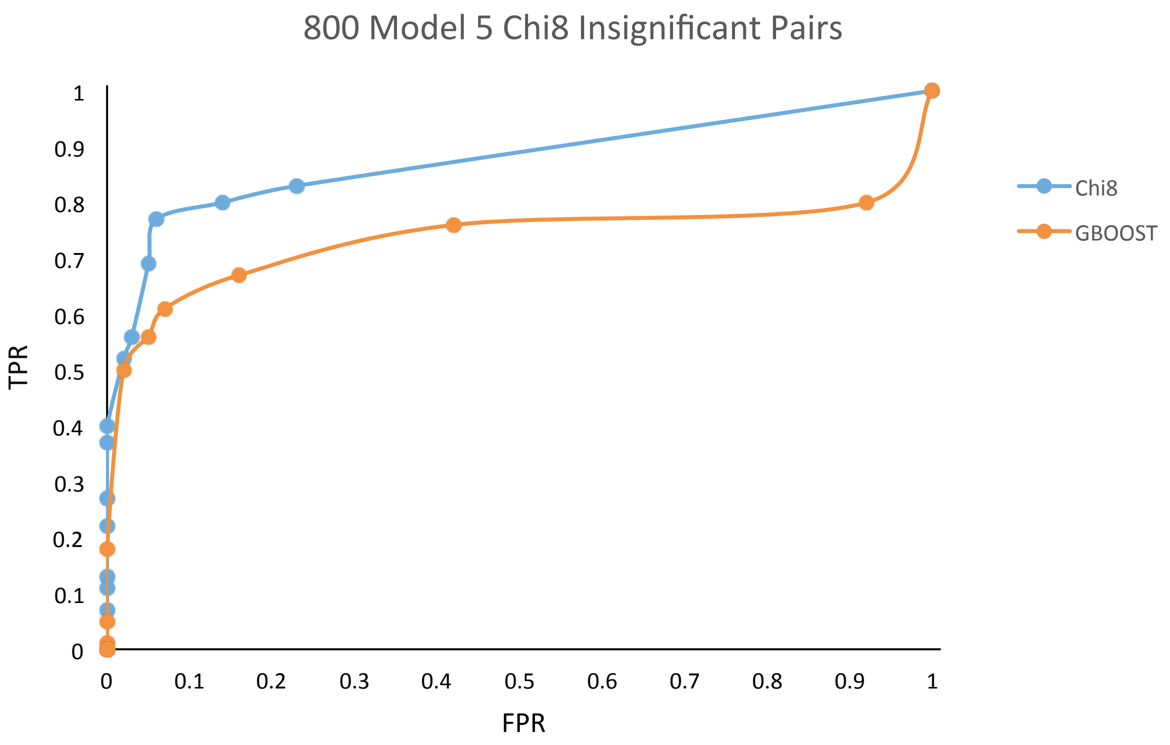
800 Model 5 Chi8 Insignificant Pairs
TPR
FPR
1
0.9
0.8
0.7
0.6
0.5
0.4
0.3
0.2
0.1
0
0.1
0.2
0.3
0.4
0.5
0.6
0.7
0.8
0.9
1
Chi8
GBOOST

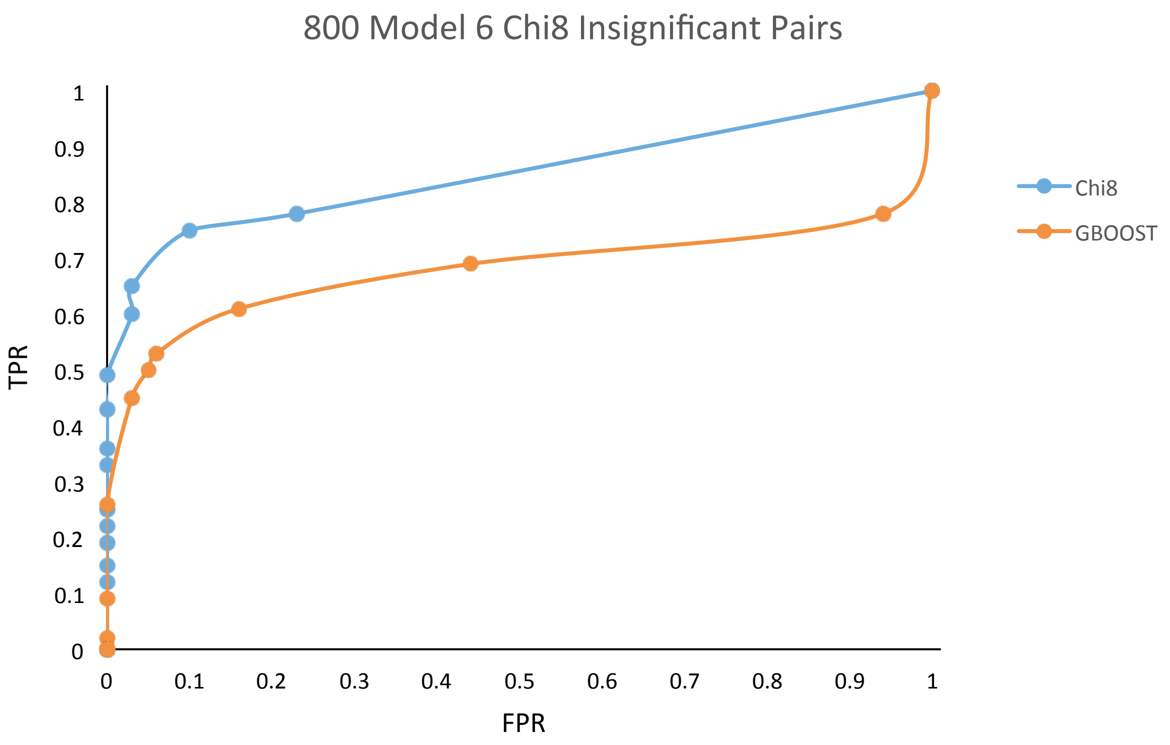

800 Model 6 Chi8 Insignificant Pairs
TPR
FPR
0
0.1
0.2
0.3
0.4
0.5
0.6
0.7
0.8
0.9
1
Chi8
GBOOST

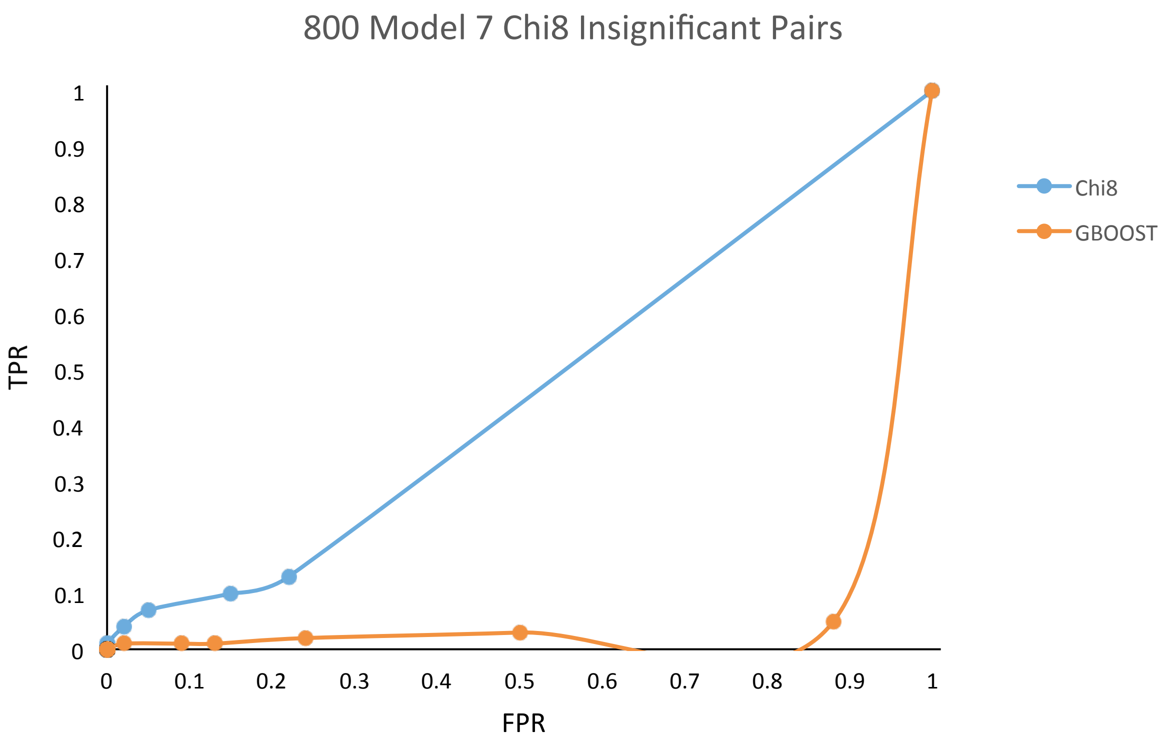
800 Model 7 Chi8 Insignificant Pairs
TPR
FPR
0
0.1
0.2
0.3
0.4
0.5
0.6
0.7
0.8
0.9
1
Chi8
GBOOST

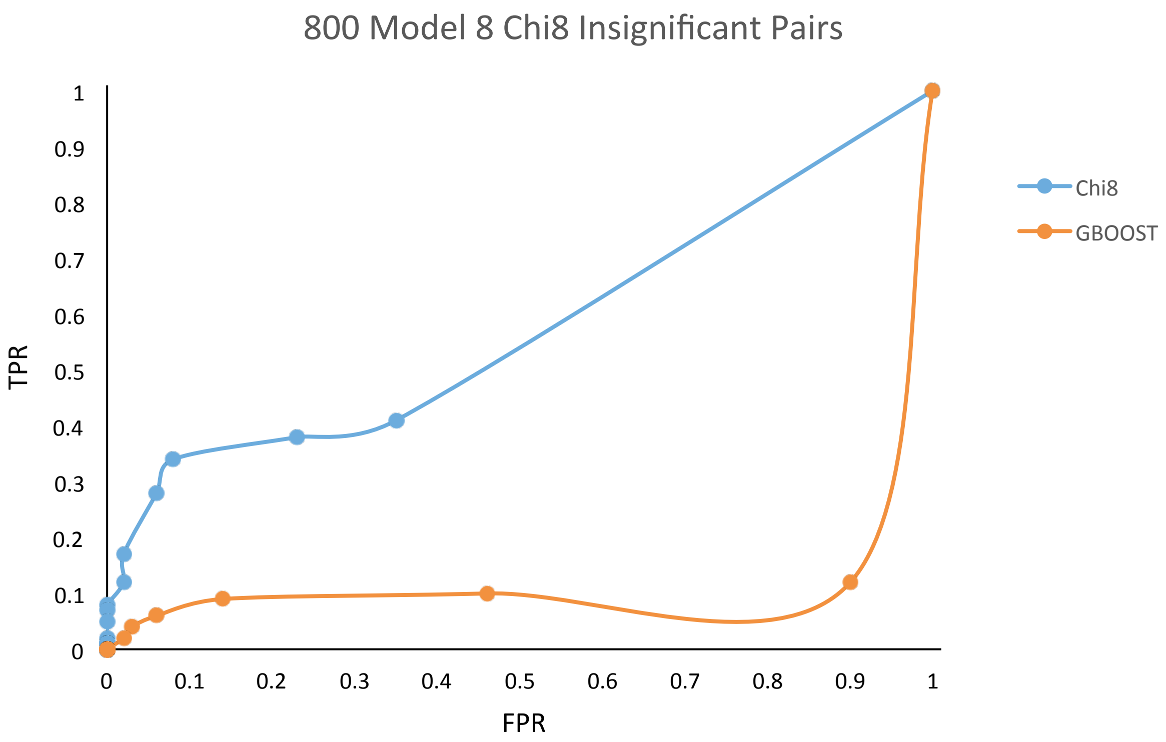
800 Model 8 Chi8 Insignificant Pairs
TPR
FPR
0
0.1
0.2
0.3
0.4
0.5
0.6
0.7
0.8
0.9
1
Chi8
GBOOST

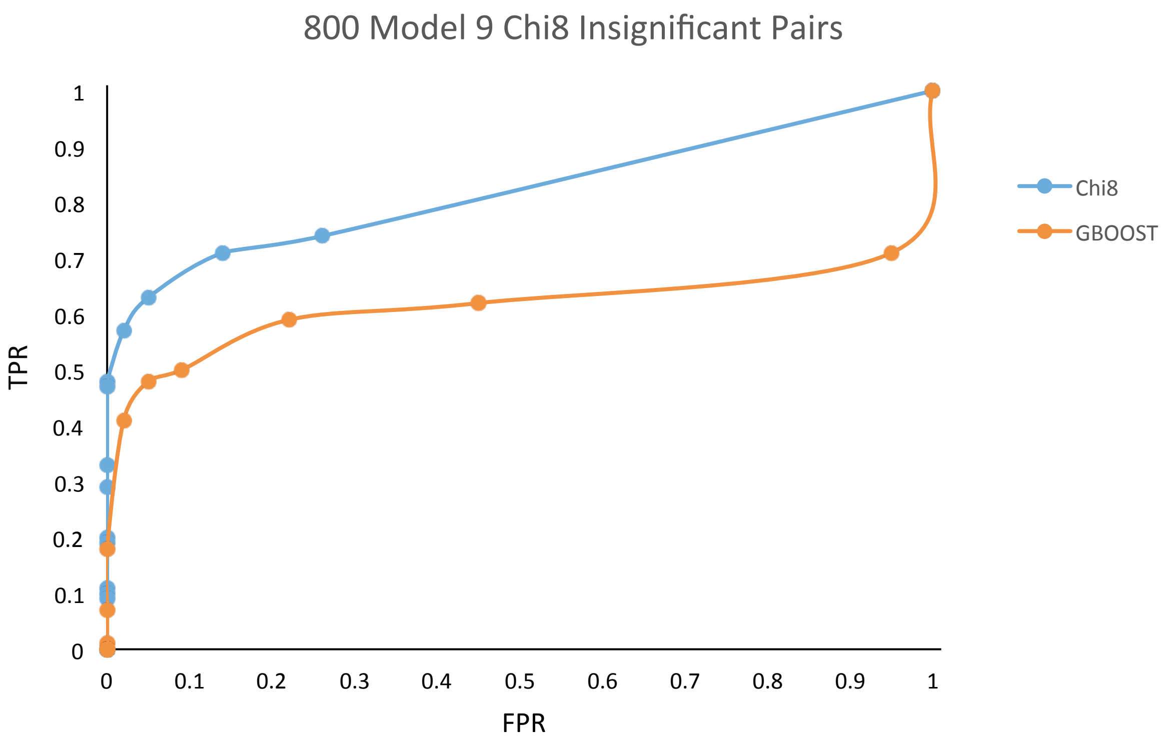
800 Model 9 Chi8 Insignificant Pairs
TPR
FPR
1
0.9
0.8
0.7
0.6
0.5
0.4
0.3
0.2
0.1
0
0
0.1
0.2
0.3
0.4
0.5
0.6
0.7
0.8
0.9
1
Chi8
GBOOST

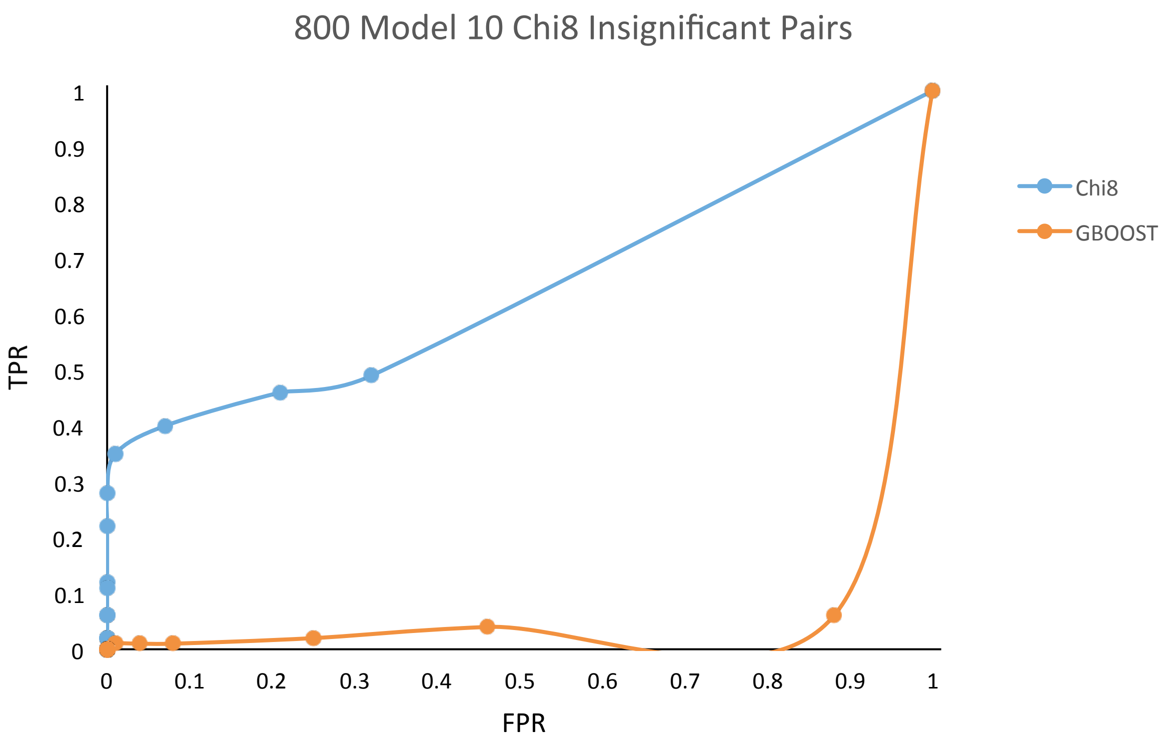

800 Model 10 Chi8 Insignificant Pairs
TPR
1
0.9
0.8
0.7
0.6
0.5
0.4
0.3
0.2
0.1
0
0
0.1
0.2
0.3
0.4
0.5
0.6
0.7
0.8
0.9
1
FPR
Chi8
GBOOST

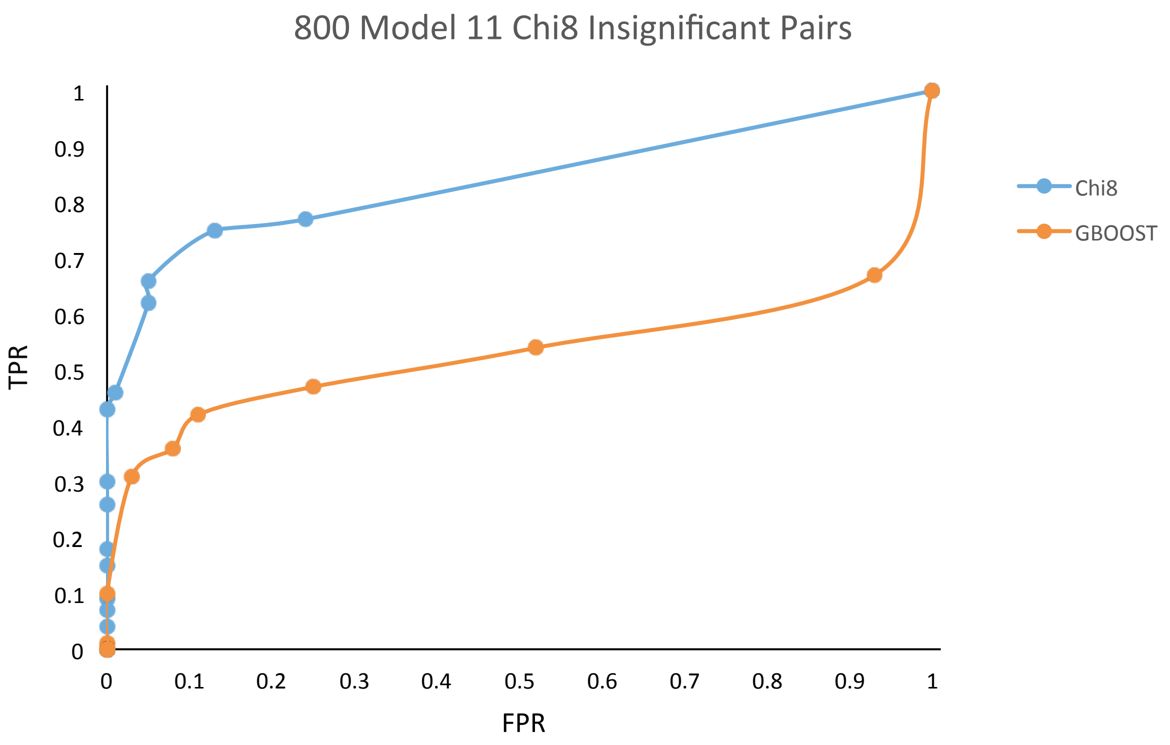
800 Model 11 Chi8 Insignificant Pairs
TPR
FPR
0
0.1
0.2
0.3
0.4
0.5
0.6
0.7
0.8
0.9
1
Chi8
GBOOST

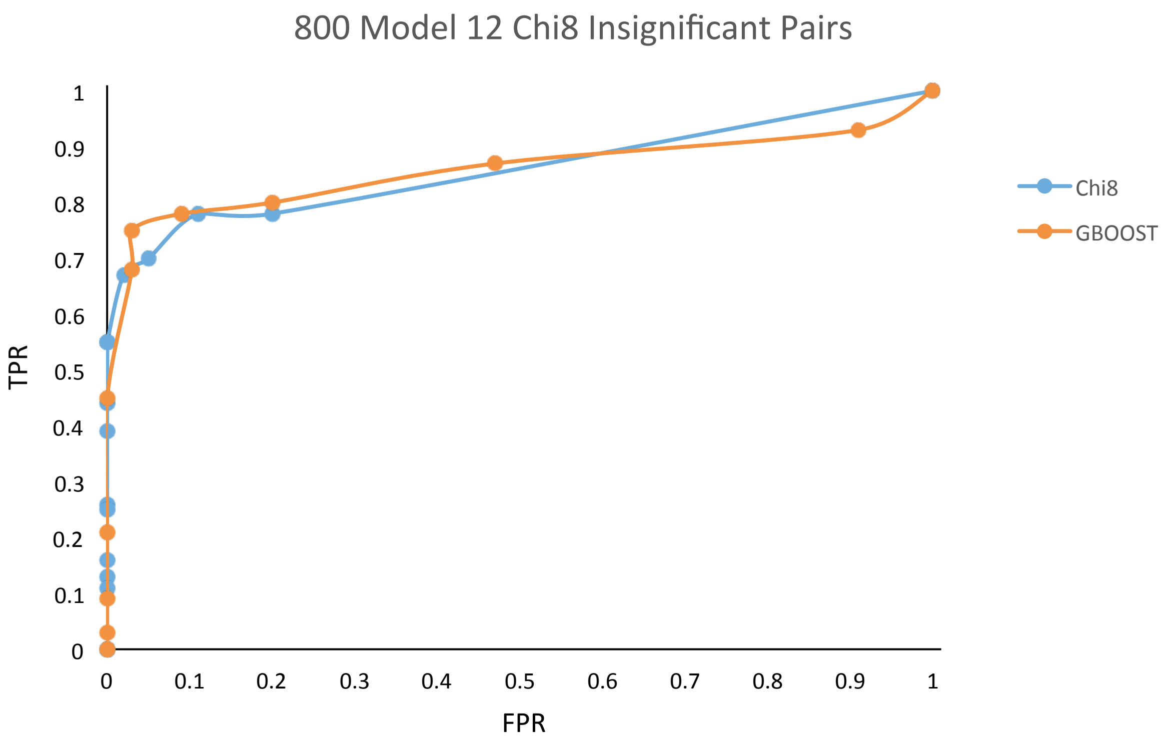

800 Model 12 Chi8 Insignificant Pairs
TPR
1
0.9
0.8
0.7
0.6
0.5
0.4
0.3
0.2
0.1
0
0
0.1
0.2
0.3
0.4
0.5
0.6
0.7
0.8
0.9
1
FPR
Chi8
GBOOST

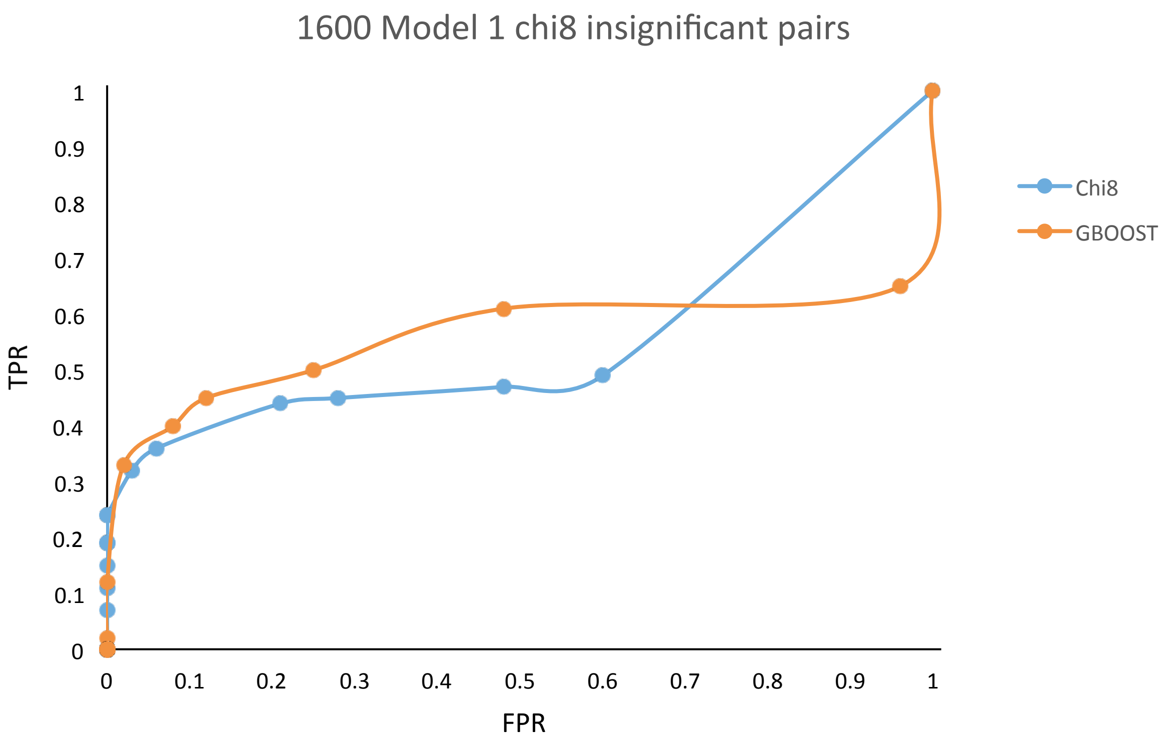
1600 Model 1 chi8 insignificant pairs
TPR
FPR
0
0.1
0.2
0.3
0.4
0.5
0.6
0.7
0.8
0.9
1
Chi8
GBOOST

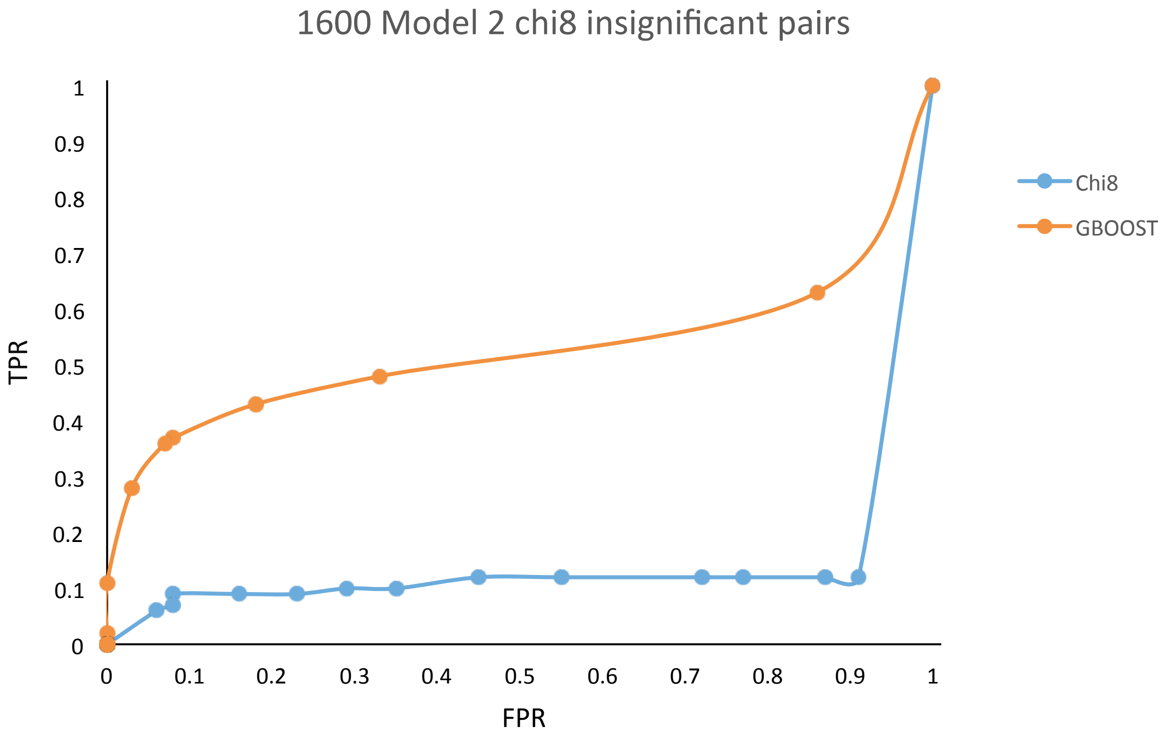

1600 Model 2 chi8 insignificant pairs
TPR
1
0.9
0.8
0.7
0.6
0.5
0.4
0.3
0.2
0.1
0
0
0.1
0.2
0.3
0.4
0.5
0.6
0.7
0.8
0.9
1
FPR
Chi8
GBOOST

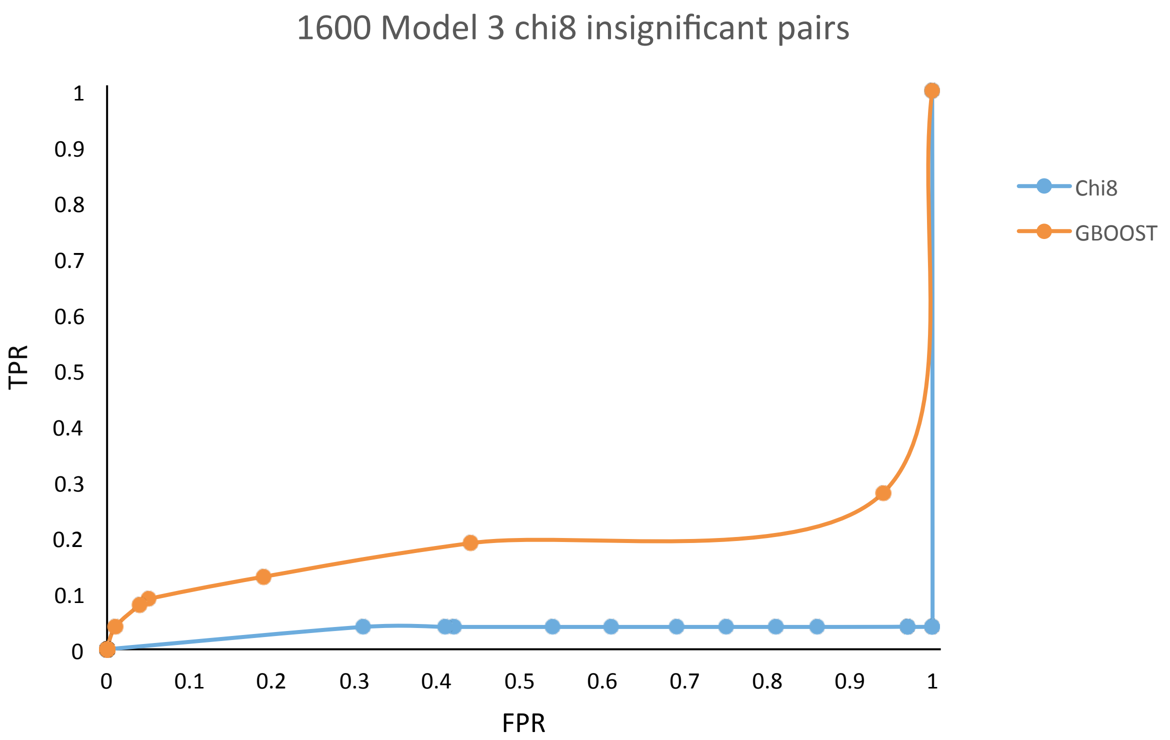
1600 Model 3 chi8 insignificant pairs
TPR
FPR
0
0.1
0.2
0.3
0.4
0.5
0.6
0.7
0.8
0.9
1
Chi8
GBOOST

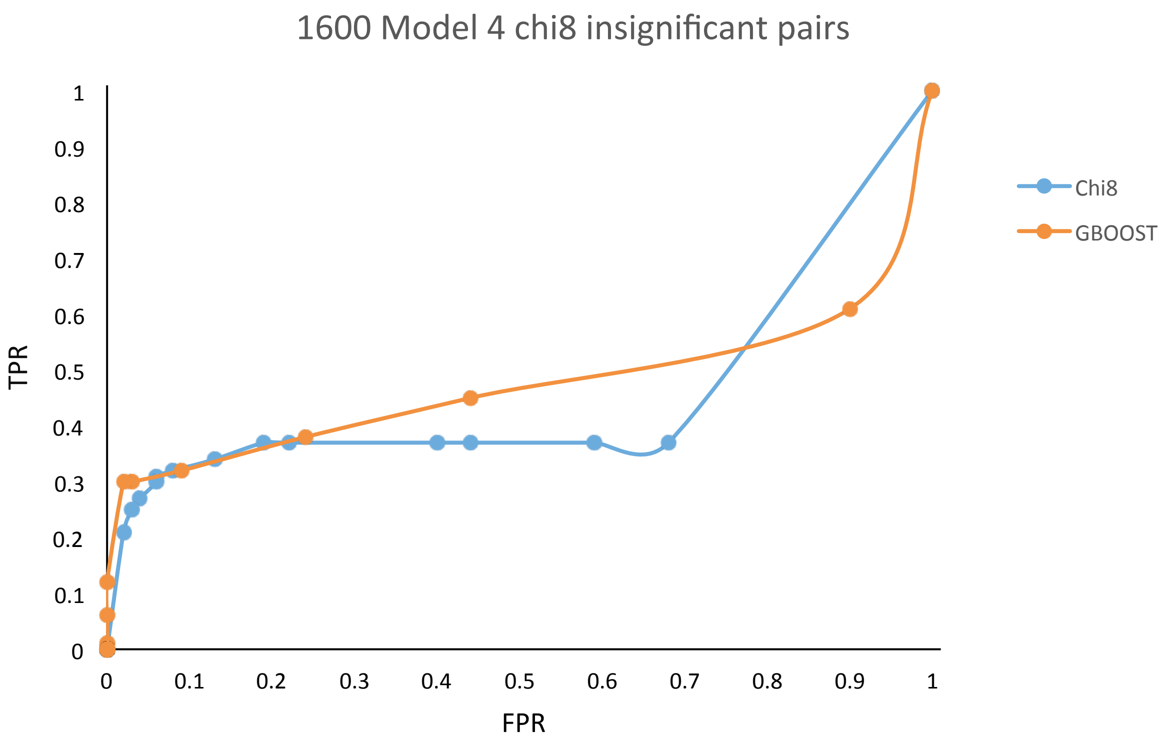
1600 Model 4 chi8 insignificant pairs
TPR
FPR
0
0.1
0.2
0.3
0.4
0.5
0.6
0.7
0.8
0.9
1
Chi8
GBOOST

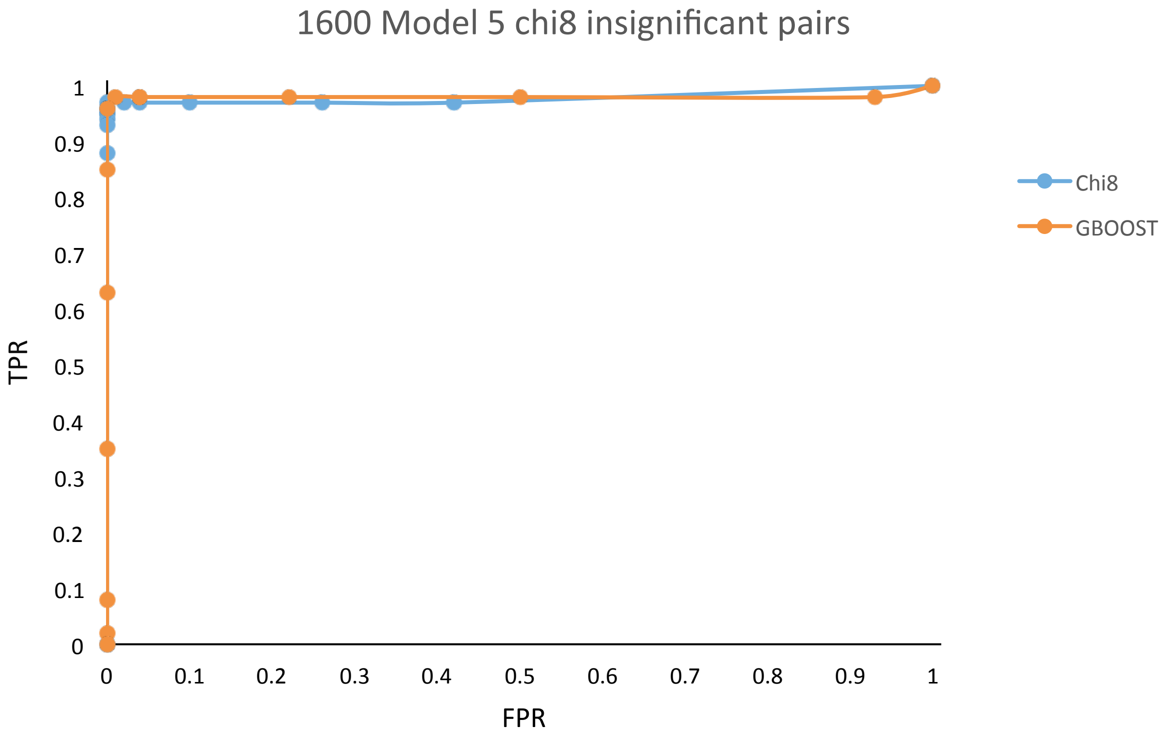
1600 Model 5 chi8 insignificant pairs
TPR
FPR
1
0.9
0.8
0.7
0.6
0.5
0.4
0.3
0.2
0.1
0
0.1
0.2
0.3
0.4
0.5
0.6
0.7
0.8
0.9
Chi8
GBOOST

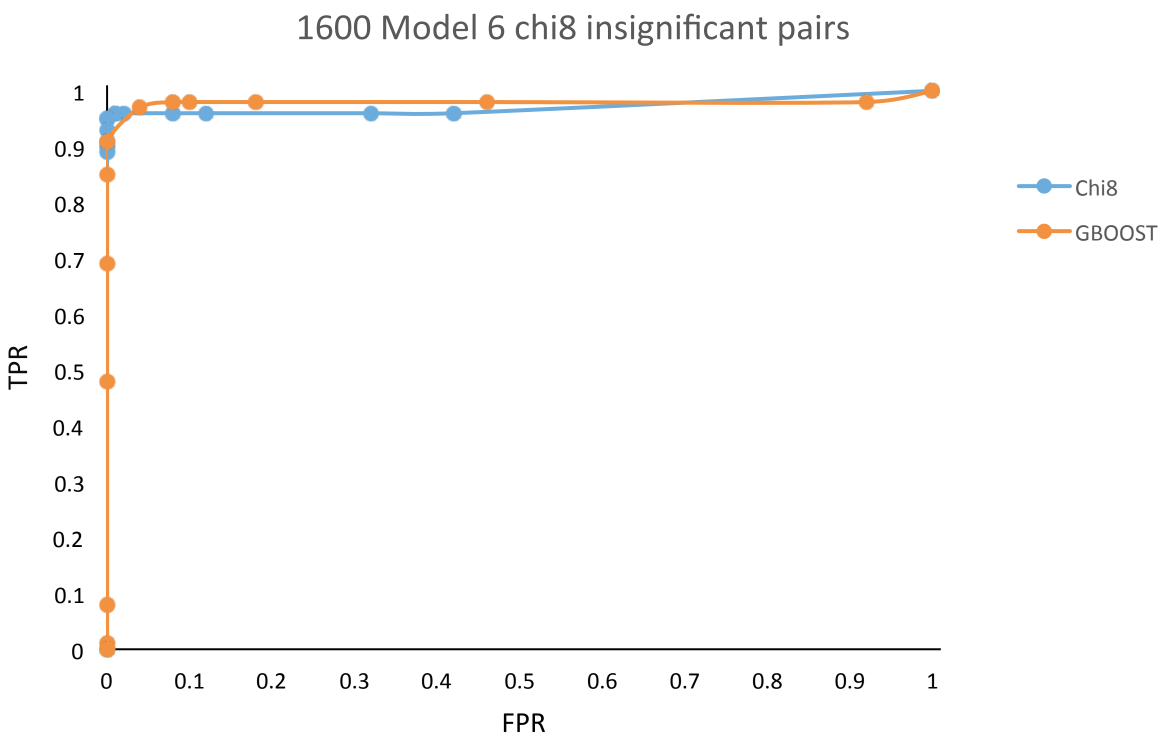

1600 Model 6 chi8 insignificant pairs
TPR
FPR
1
0.9
0.8
0.7
0.6
0.5
0.4
0.3
0.2
0.1
0
0
0.1
0.2
0.3
0.4
0.5
0.6
0.7
0.8
0.9
1
Chi8
GBOOST

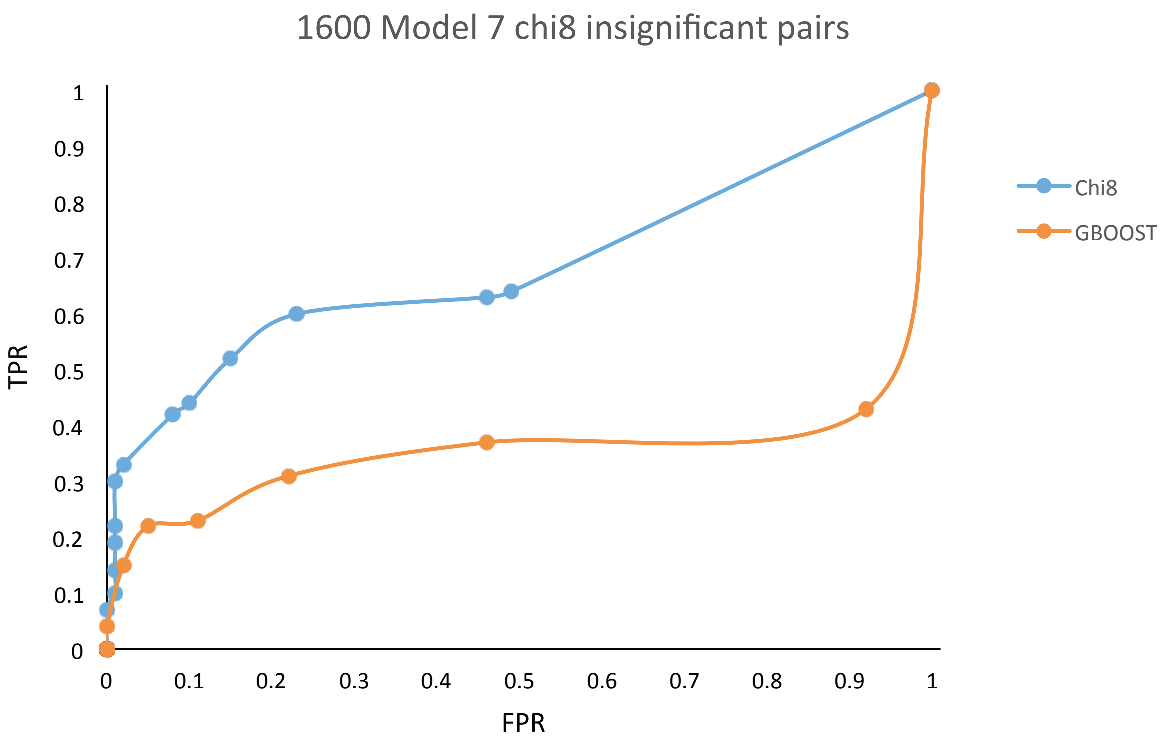
1600 Model 7 chi8 insignificant pairs
TPR
FPR
0
0.1
0.2
0.3
0.4
0.5
0.6
0.7
0.8
0.9
1
Chi8
GBOOST

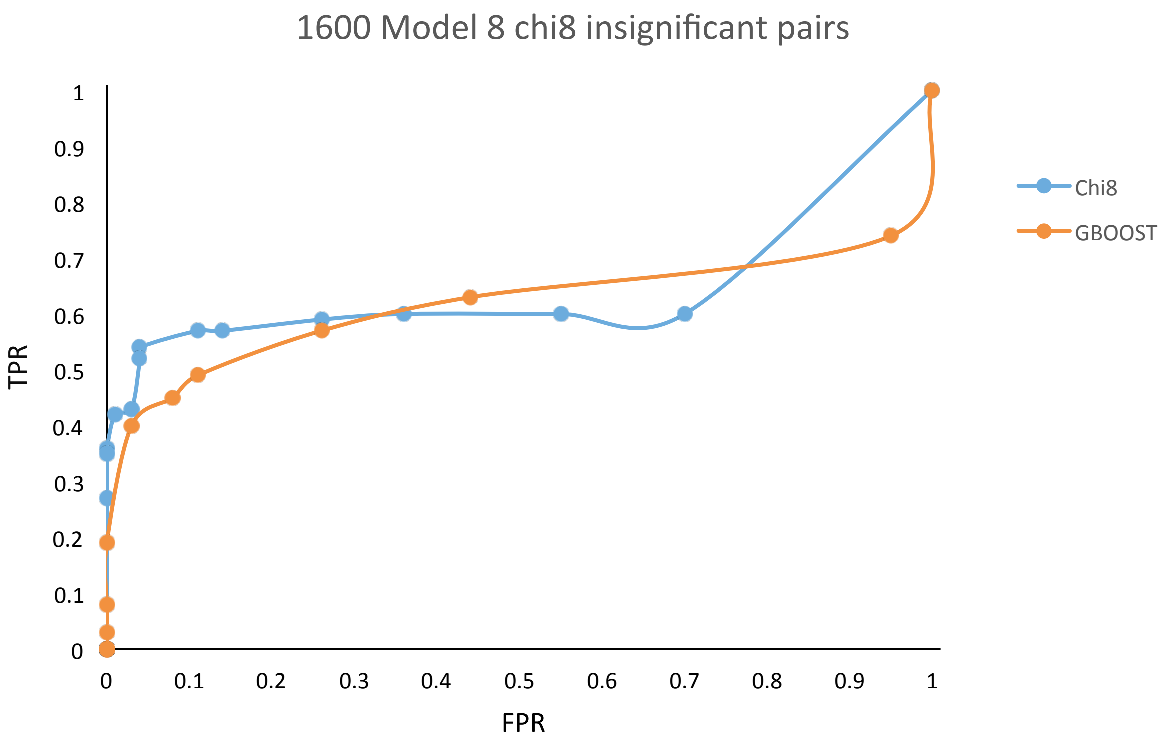

1600 Model 8 chi8 insignificant pairs
TPR
FPR
1
0.9
0.8
0.7
0.6
0.5
0.4
0.3
0.2
0.1
0
0
0.1
0.2
0.3
0.4
0.5
0.6
0.7
0.8
0.9
1
Chi8
GBOOST

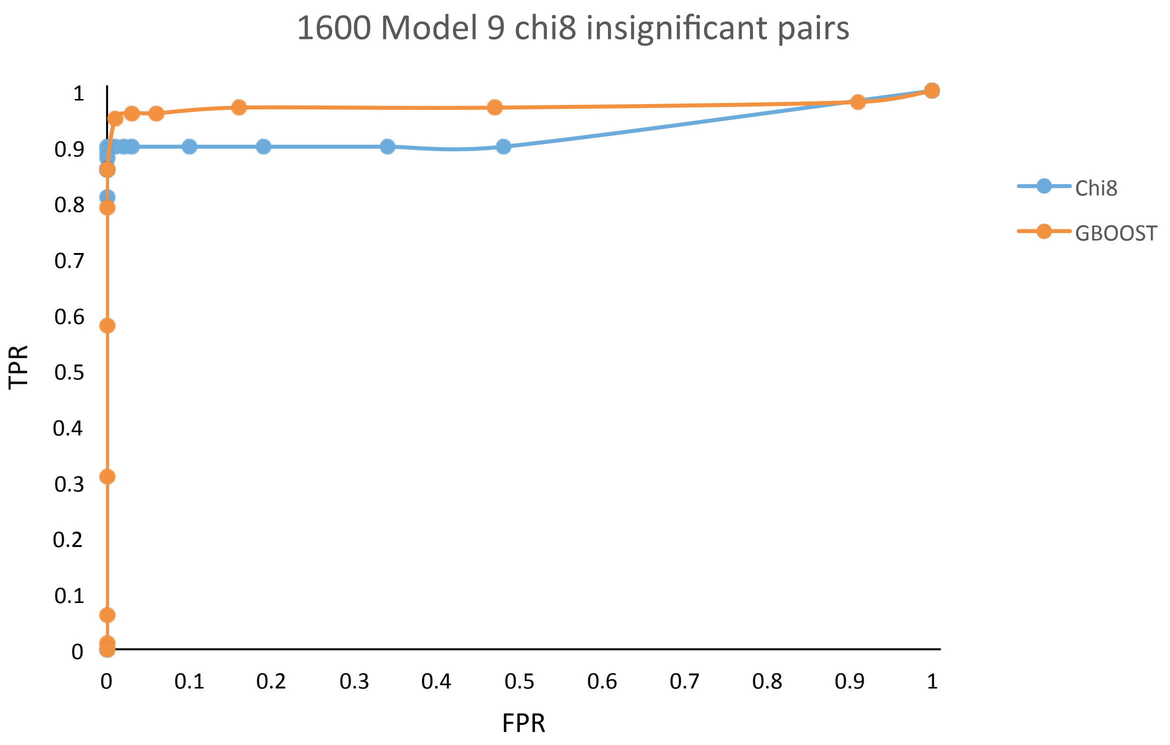

1600 Model 9 chi8 insignificant pairs
TPR
FPR
1
0.9
0.8
0.7
0.6
0.5
0.4
0.3
0.2
0.1
0
0.1
0.2
0.3
0.4
0.5
0.6
0.7
0.8
0.9
1
Chi8
GBOOST

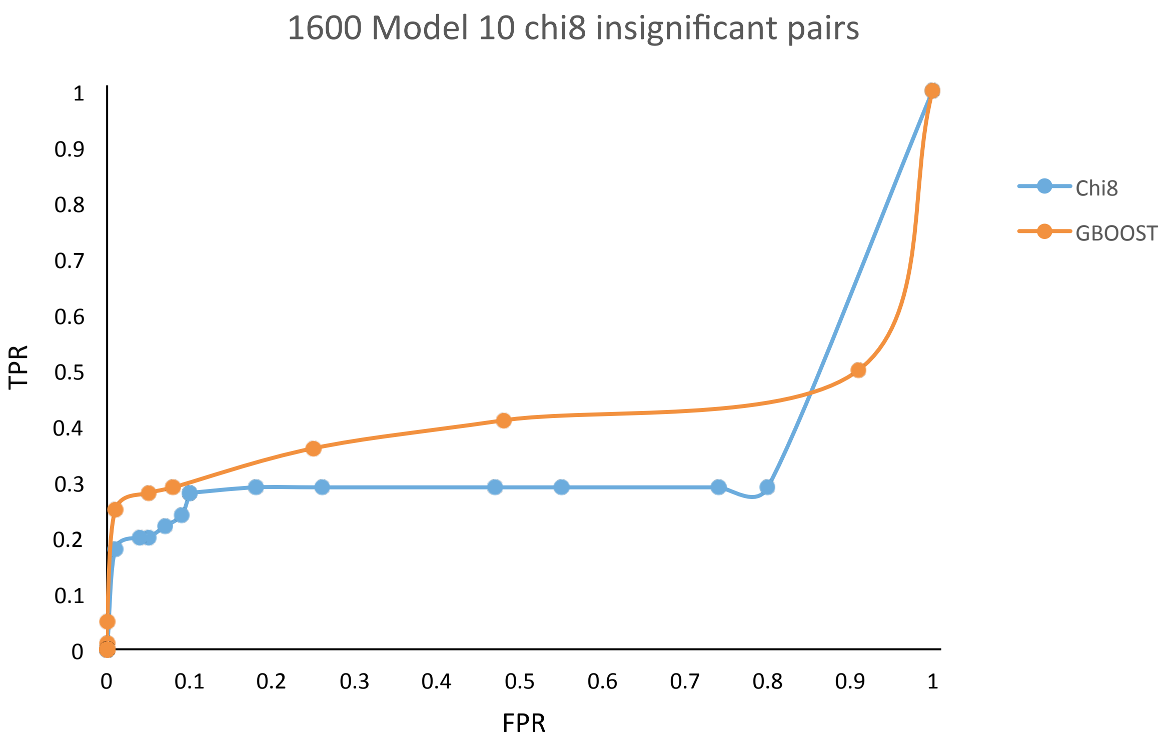

1600 Model 10 chi8 insignificant pairs
TPR
FPR
1
0.9
0.8
0.7
0.6
0.5
0.4
0.3
0.2
0.1
0
0
0.1
0.2
0.3
0.4
0.5
0.6
0.7
0.8
0.9
1
Chi8
GBOOST

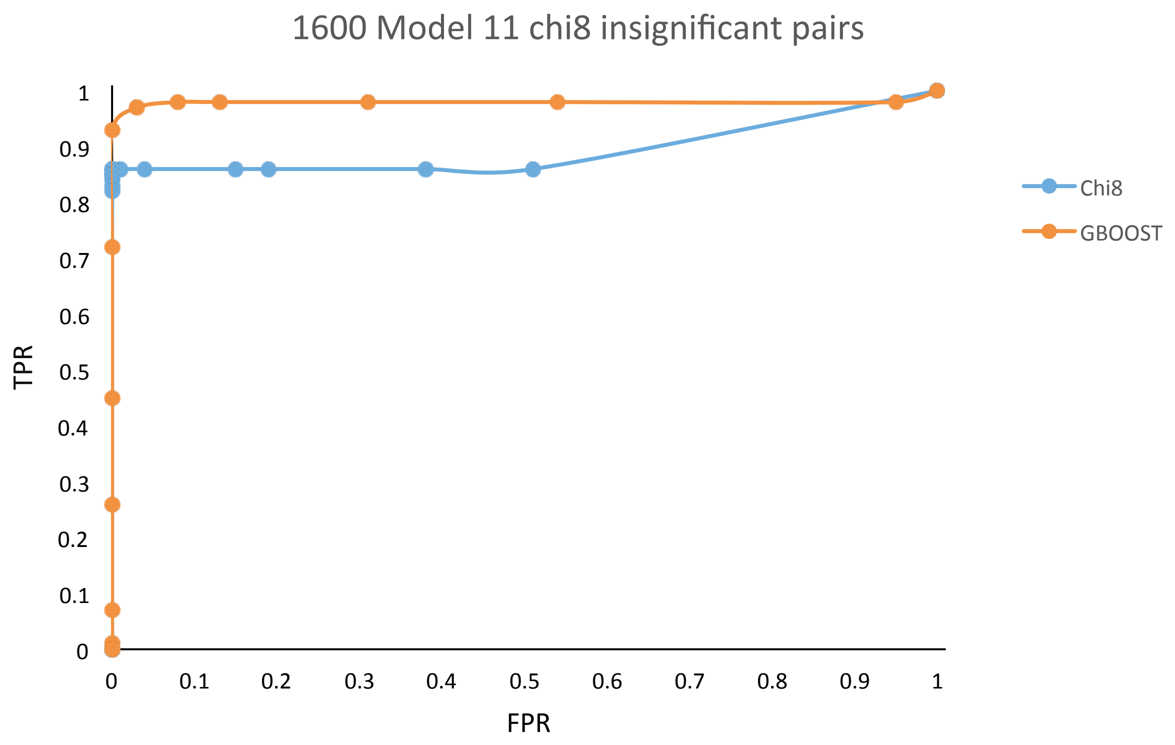
1600 Model 11 chi8 insignificant pairs
TPR
FPR
0
0.1
0.2
0.3
0.4
0.5
0.6
0.7
0.8
0.9
1
Chi8
GBOOST

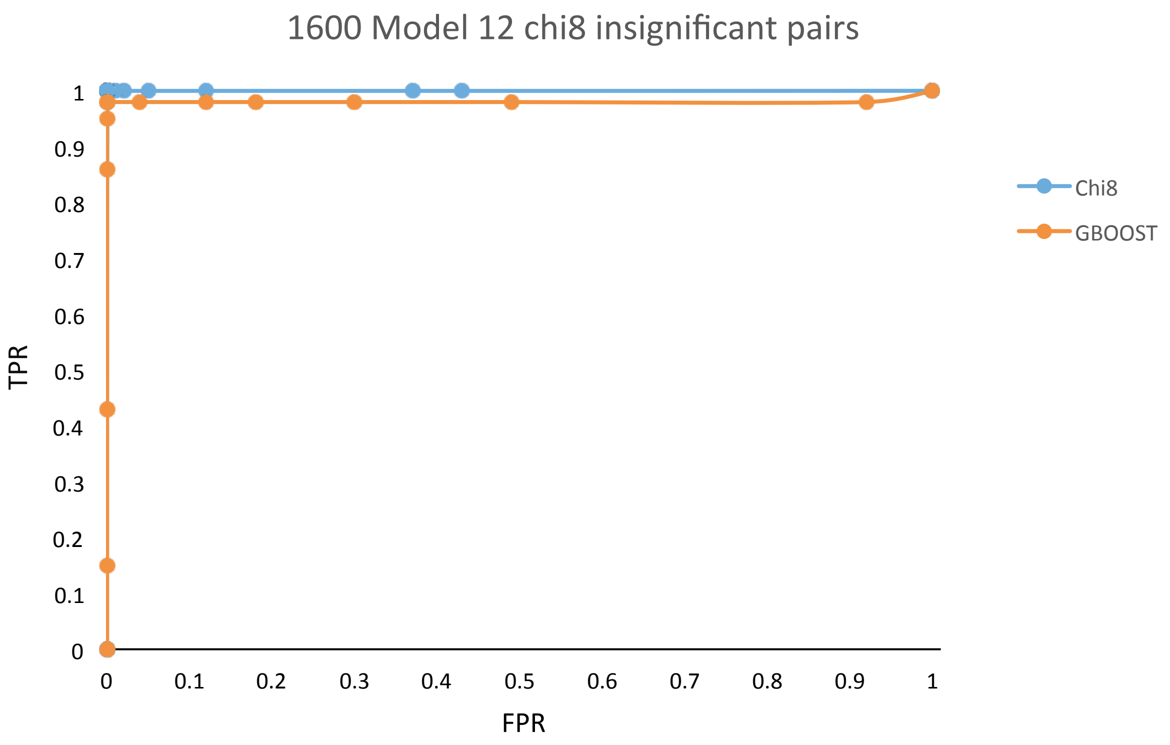

1600 Model 12 chi8 insignificant pairs
TPR
1
0.9
0.8
0.7
0.6
0.5
0.4
0.3
0.2
0.1
0
0
0.1
0.2
0.3
0.4
0.5
0.6
0.7
0.8
0.9
1
FPR
Chi8
GBOOST
